# Rupintrivir reduces RV-induced T_H_-2 cytokine IL-4 in precision-cut lung slices (PCLS) of HDM-sensitized mice ex vivo

**DOI:** 10.1186/s12931-019-1175-y

**Published:** 2019-10-22

**Authors:** Olga Danov, Lisa Lasswitz, Helena Obernolte, Christina Hesse, Armin Braun, Sabine Wronski, Katherina Sewald

**Affiliations:** 1 Fraunhofer Institute for Toxicology and Experimental Medicine ITEM, Biomedical Research in Endstage and Obstructive Lung Disease Hannover (BREATH), Member of the German Center for Lung Research (DZL), Member of Fraunhofer International Consortium for Anti-Infective Research (iCAIR), Nikolai-Fuchs-Strasse 1, 30625 Hannover, Germany; 2 Institute of Immunology, Hannover Medical School, Carl-Neuberg Strasse 1, 30625 Hannover, Germany

**Keywords:** Rhinovirus, Infection, Asthma, Exacerbation, Lung sections

## Abstract

**Background:**

Antiviral drugs such as rupintrivir may have an immune-modulatory effect in experimentally induced allergic asthma with subsequent RV infection. We infected lung slices of house-dust mite (HDM)-sensitized asthmatic mice ex vivo with human rhinovirus (RV) and investigated the effect of the antiviral drug rupintrivir on RV-induced cytokine response in lung tissue of HDM-sensitized mice ex vivo.

**Methods:**

Mice were sensitized with HDM. Precision-cut lung slices (PCLS) were prepared from HDM-sensitized or non-sensitized mice. Lung slices were infected ex vivo with RV or RV together with rupintrivir. Modulation of immune responses was evaluated by cytokine secretion 48 h post infection.

**Results:**

In vivo HDM sensitization resulted in a T_H_-2/T_H_-17-dominated cytokine response that persisted in PCLS ex vivo. RV infection of PCLS from non-sensitized mice resulted in the induction of an antiviral and pro-inflammatory immune response, as indicated by the secretion of IFN-α, IFN-β, IFN-γ, TNF-α, MCP-1, IP-10, IL-10, and IL-17A. In contrast, PCLS from HDM-sensitized mice showed an attenuated antiviral response, but exaggerated IL-4, IL-6, and IL-10 secretion upon infection. Rupintrivir inhibited exaggerated pro-inflammatory cytokine IL-6 and T_H_-2 cytokine IL-4 in HDM-sensitized mice.

**Conclusions:**

In summary, this study demonstrates that treatment with rupintrivir influences virus-induced IL-4 and IL-6 cytokine release under experimental conditions ex vivo.

**Electronic supplementary material:**

The online version of this article (10.1186/s12931-019-1175-y) contains supplementary material, which is available to authorized users.

## Background

Upper respiratory tract infections, such as the common cold, are usually caused by viruses and are self-limiting in healthy individuals. In patients with chronic pulmonary diseases such as asthma and chronic obstructive pulmonary disease (COPD), human rhinovirus (RV) infections can be more severe and associated with a decline in lung function. Several studies have demonstrated dysregulated host antiviral immune responses in asthmatic patients, leading to viral transmission from the upper to the lower respiratory tract. A disrupted epithelial barrier and attenuated elimination of viruses by ciliary dysfunction results in stronger viral propagation [1–4], which finally can lead to morbidity and hospitalization [5–7].

Treatments for asthmatic patients are mainly based on inhaled corticosteroids and long-acting β2-adrenoceptor agonists, however, these drugs do not prevent viral-induced exacerbations. Several specific antiviral treatments, targeting infection, are currently under development for effective clinical management and reduced morbidity.

Rupintrivir (AG7088, developed by Agouron Pharmaceuticals) is a synthetic 3C protease inhibitor, with potent in vitro activity against viral serotypes [8]. The 3C protease is essential for cleaving the polyprotein precursor into structural proteins and enzymes that are required for viral replication [9]. In in vitro cell cultures of BEAS-2B (an immortalized human bronchial epithelial cell line) rupintrivir has shown potent antiviral activity as well as reduced RV-induced interleukin (IL)-6 and IL-8 release compared with untreated cells [10]. During a phase-II clinical trial, rupintrivir failed to show beneficial effects in patients with naturally acquired colds with no underlying chronic respiratory disease [11]. In this double-blinded clinical trial, rupintrivir was applied within 36 h of cold symptoms emergence, and showed only moderate efficacy. Although antiviral drugs against the common cold failed to relieve symptoms in healthy individuals, patients with prolonged chronic lung diseases may benefit from a drug preventing symptom amplification. The effect of rupintrivir in allergic asthma is unknown and has not been investigated so far. We hypothesize that the effect of the antiviral drug rupintrivir is altered under asthmatic conditions and may result in beneficial effects in prevention of exaggerated T_H_-2 immune responses.

## Materials and methods

### Media, chemicals, and reagents

For cell culture, minimal essential medium (MEM) was supplemented with 100 units/mL penicillin and streptomycin, 2 mM L-glutamine, and 1 × NEAA (Non-Essential Amino Acids Solution), all purchased from Gibco (Life Technologies, Darmstadt, Germany). Dulbecco’s phosphate-buffered solution without Ca^2**+**^ and Mg^2**+**^ (DPBS) was purchased from Lonza (Verviers, Belgium). Low-gelling temperature agarose, protease inhibitor cocktail P1860, Triton X-100, and Earle’s Balanced Salt Solution (EBSS) were purchased from Sigma-Aldrich (Munich, Germany). The Pierce BCA protein assay kit was supplied by Thermo Scientific (Rockford, IL, USA). Human rhinovirus 1b was obtained from Virapur (Lot J1323A, San Diego, CA, USA). Rupintrivir was purchased from Axon Medchem BV (AG7088, Groningen, The Netherlands). Purified HDM extract from *Dermatophagoides pteronyssinus* was obtained from GREER (Lenoir, NC, USA).

### Animals and husbandry conditions

Female mice (Balb/c, 6–8 weeks) were obtained from Charles River (Sulzfeld, Germany) and kept under conventional housing conditions (22 °C, 55% humidity, and 12 h day/night rhythm).

### HDM-induced chronic allergic airway inflammation

Balb/c mice were sensitized by intranasal application of 25 μg HDM in 50 μL of saline (control group received saline only) for four days per week, over four weeks as previously published [12], and used for the preparation of PCLS.

### BAL differential cell count

The BAL fluid was centrifuged (320 g, 10 min, 4 °C) and the supernatant immediately frozen at − 80 °C. Total cell count of cells resuspended in 0.5 ml of PBS was performed automatically with a Casy® cell counter. Cytospots were stained by QuickDiff (Medion Diagnostic, Duedingen, Switzerland) to evaluate differential cell counts.

### Preparation of PCLS

Precision-cut lung slices (PCLS) were prepared from HDM-sensitized (abbreviated as “sensitized”) or saline-treated mice (abbreviated as “non-sensitized”) as described previously [13, 14]. Briefly, lungs were inflated using agarose/medium solution. After polymerization, slices of 350 μm were cut in 4 °C cold EBSS, using an automatic oscillating tissue slicer (OTS 5000, Warner Instruments, CT, USA). Tissue slices were transferred into a medium-filled petri dish and incubated under cell culture conditions (37 °C, and 5% CO_2_). The medium was exchanged at least four times every 30 min for 2–3 h to remove cell debris. Only PCLS containing an airway with an intact smooth muscle layer and active beating of the cilia were selected for the study.

From each mouse lung, a sufficient number of PCLS were generated to test control, RV, UV-inactivated RV, and RV + Rup conditions in duplicates with 2 PCLS per well. The approach was repeated with 5 mice (*n* = 5) per group.

### Viral titer

The viral titer (tissue culture infection dose 50, TCID_50_) of the Virapur RV1b stock was measured by limiting dilution plating on HeLa Ohio cells (Health Protection Agency, #84121901), and determining the cytopathic effect as previously described [12]. The TCID_50_ value of the stock was 1.05 × 10^7^ IU/mL (infectious units per mL).

### RV infection of PCLS

Two PCLS per well were infected with 1 × 10^5^ IU/mL of RV for 48 h in culture medium at cell culture conditions (33 °C, 5% CO_2_, and 100% humidity). To determine the replication dependent immune response, UV-inactivated virus (260 nm for 1 h) from the same batch was used under the same conditions. Pharmacological intervention was performed by infection with RV (1 × 10^5^ IU/mL) in the presence of 100 nM rupintrivir.

After incubation for 48 h, the tissue culture supernatants were collected. For the analysis of intrinsic cytokines, PCLS were permeabilized with 1% Triton X-100 in DPBS for 1 h at 4 °C. All samples were supplemented with 0.2% protease inhibitor cocktail and stored at − 80 °C for further analysis.

### Protein determination

The total protein amount of the samples was determined using the Pierce BCA protein assay kit (ThermoFisher Scientific, Rockford, IL, USA) according to the manufacturer’s instructions as described previously [15].

### Measurement of pro-inflammatory and antiviral cytokines (ELISA/MSD)

Mouse IFN-α, IFN-β, IL-6, MCP-1, and IP-10 were quantified using commercially available enzyme-linked immunosorbent assay kits (ELISA Duosets, R&D Systems, Wiesbaden, Germany) according to the manufacturer’s instructions. IFN-γ, IL-4, IL-5, IL-10, IL-13, IL-17A, IL-17E/IL-25, IL-17F, IL-33, and TNF-α were measured using a mouse U-PLEX Biomarker Group 1 (ms) assay from Meso Scale Discovery (MSD; Gaithersburg, MD, USA) according to the manufacturer’s instructions, and measured with a MSD Sector Imager 2400. Cytokine concentrations were calculated based on a 4-fold serially diluted standard. Data analysis was conducted using the Discovery Workbench software. The cytokine content of each sample was related to the total protein content (pg cytokine/mg total protein).

### Calcein AM/ethidium homodimer-1 (LIVE/DEAD®) staining and quantitative image analysis

Viability of PCLS was assessed by Calcein AM/ethidium homodimer-1 staining as described before [15]. From each PCLS, triplicates of 30 μm thick 3D stacks were analyzed using IMARIS 7.4.0 software (Bitplane Scientific Software, Zurich, Switzerland), as described previously [15]. The ratio of counted dead cell nuclei (ethidium homodimer-1 positive) to total volume of cytoplasm of living cells (calcein stained) was calculated as dead cell nuclei/10^6^ μm^3^ cytoplasm volume.

### Statistical analysis

Statistical analysis was performed using GraphPad Prism 8.0.1. The statistical tests used and the sample size are given in each figure legend.

## Results

### Cytokine profile of PCLS from HDM-sensitized mice

**Fig. 1:**
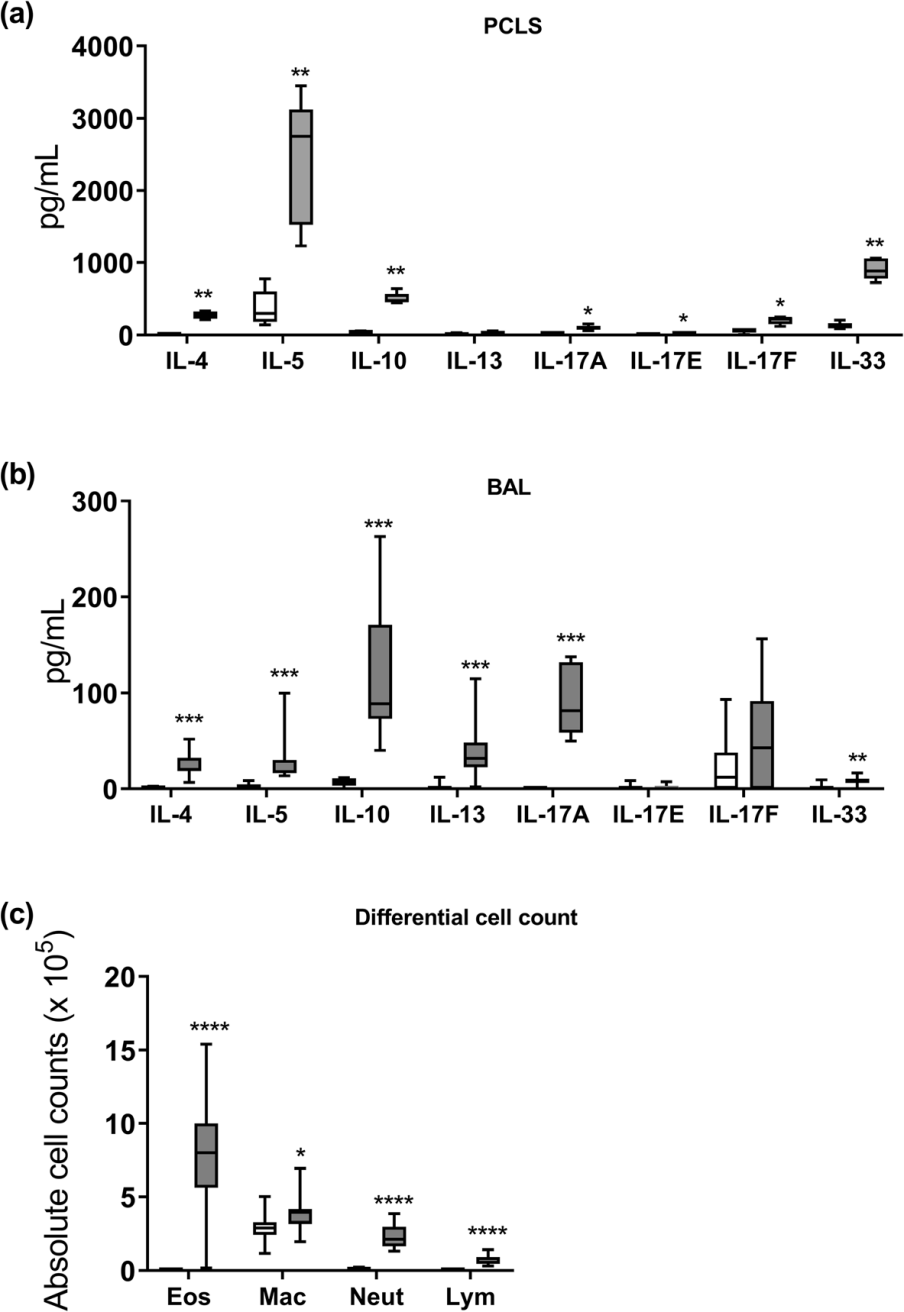
Cytokine profile of PCLS (precision-cut lung slices) from HDM (house-dust mite)-sensitized mice in comparison to cytokine profile of BAL (bronchoalveolar lavage) from HDM-sensitized mice. Balb/c mice were treated with saline (white bars) or HDM (grey bars) for four weeks. (**a**) Cytokine profile of PCLS isolated from HDM- and non-sensitized mice after 48 h ex vivo culture without any further stimulation. Data are shown as box whisker plots (mean to max), *n* = 5 mice with two technical replicates (duplicate wells with 2 PCLS each), * *p* ≤ 0.05, and ** *p* ≤ 0.01 according to Mann–Whitney U test. (**b**) BAL cytokine profile 24 h after the last HDM/saline challenge. (**c**) BAL differential cell counts for eosinophils (Eos), macrophages (Mac), neutrophils (Neut), and lymphocytes (Lym). Graphs (b)-(c) are shown as box whisker plots (mean to max), *n* = 10 animals per group, * *p* ≤ 0.05, ** *p* ≤ 0.01, *** *p* ≤ 0.001, and **** *p* ≤ 0.0001 according to the Mann–Whitney U test

Our first aim was to characterize the ex vivo cytokine profile of PCLS prepared from HDM-sensitized mice. Without any restimulation, PCLS from HDM-sensitized mice showed increased cytokine secretion ex vivo after 48 h compared to PCLS from non-sensitized mice. Cytokines IL-4 (35-fold) and IL-10 (8.3-fold) were increased, whereas IL-5 (5.2-fold), IL-17A (2.6-fold), IL-17E (3.2-fold), IL-17F (2.1-fold), and IL-33 (5.4-fold) were only slightly increased and IL-13 remained unaltered (Fig. 1a). To verify that the cytokine profile measured ex vivo was comparable to the in vivo phenotype, the data were compared to BAL (bronchoalveolar lavage) cytokine profiles of different animals of the same group that were equally exposed to HDM or saline. In BAL fluid, elevated cytokine levels were observed for IL-4 (20-fold), IL-5 (8.9-fold), IL-10 (18-fold), IL-13 (19-fold), IL-17A (303-fold), and IL-33 (4-fold), whereas IL-17E and IL-17F levels remained unchanged (Fig. 1b). Furthermore, BAL differential cell counts of HDM-sensitized mice confirmed airway inflammation indicated by increased infiltration of eosinophils, macrophages, neutrophils, and lymphocytes compared to non-sensitized mice (Fig. 1c).

### Rupintrivir modulates exaggerated IL-4 and IL-6 inflammation in lung tissue of HDM-sensitized mice

To gain first insight about the efficacy of rupintrivir in lower respiratory tract infections under asthmatic conditions, the cytokine response to RV infection and the effect of rupintrivir on this response were analyzed ex vivo in lung tissue of HDM-sensitized and non-sensitized mice. Viability of PCLS was not affected throughout the infection experiment as confirmed by confocal imaging of calcein AM / ethidiumhomodimer-1 (EthD-1) stained slices (Additional file 1 (a)-(d)). Quantification of the analyzed images showed that the ratio of dead cells to volume of living cells remains constant for the entire period of time (Additional file 1 (e)).

PCLS from HDM-sensitized and non-sensitized mice were infected with RV for 48 h. Infection caused a slight accumulation of dead cells around airways although that increase was not sufficient to change the total number of dead cells. The viral load in tissue culture supernatant was quantified 48 h p.i. as infectious units (IU) assessed by dilution plating on HeLa cells and calculation of tissue culture infective dose 50 (TCID_50_) based on observation of cytopathic effects. The virus load detected in infected tissue was not changed in comparison to the initial inoculum of 10^5^ IU/mL. Similar results were obtained by PCR (data not shown). Each lung slice contained only one cross-sectioned airway with a diameter of about 500 μm. Accordingly, only one circular layer of airway epithelial cells lining this airway can be infected by RV. Consequently, as the total number of airway epithelial cells in lung tissue sections ex vivo is very low (compared to conventional cell culture infections), and not all airway epithelial cells get infected, the number of virions released from those cells is also low (data not shown). Thus, any additional increase of virus load based on active viral replication in these few cells was not detectable by TCID_50_ or PCR beyond the initial virus amount present in supernatant of lung slices under these experimental conditions.

**Fig. 2:**
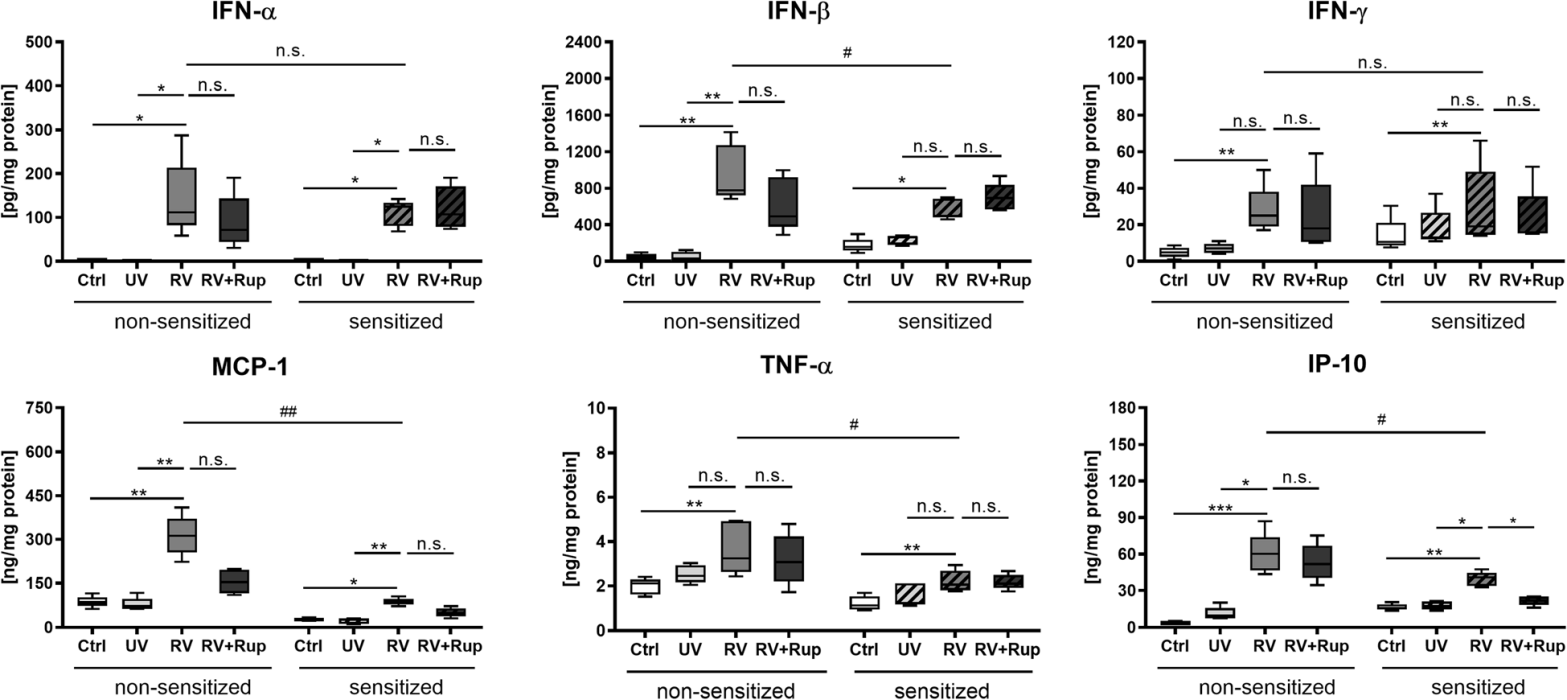
Antiviral cytokine response to RV (rhinovirus) infection is impaired in PCLS of HDM-sensitized mice and shows limited response to rupintrivir treatment. PCLS from non-sensitized and HDM-sensitized mice were infected with 10^5^ TCID_50_/mL of UV-inactivated RV (UV) or RV, or RV in combination with 100 nM rupintrivir (Rup) for 48 h. Uninfected slices were used as a control (Ctrl). All data are shown as box whisker plots (mean to max), *n* = 5 mice with two technical replicates (duplicate wells with 2 PCLS each), statistical analysis performed for non-sensitized or HDM-sensitized group with * *p* ≤ 0.05; ** *p* ≤ 0.01; *** *p* ≤ 0.001; **** *p* ≤ 0.0001; according to Friedman with Dunn’s multiple comparison post-hoc test. Means of RV infected PCLS from non-sensitized and HDM-sensitized animals were considered statistically significant with # *p* ≤ 0.05 and ## *p* ≤ 0.01 according to the Mann–Whitney U test. n.s. = not significant

PCLS isolated from non-sensitized mice showed a strong antiviral response to active viral infection, which resulted in enhanced secretion of IFN-α (0.4 vs. 112 pg/mg), IFN-β (31 vs. 779 pg/mg), IFN-γ (7 vs. 25 pg/mg), MCP-1 (73 vs. 313 ng/mg), TNF-α (2.5 vs. 3.2 ng/mg), and IP-10 (9 vs. 60 ng/mg) (Fig. 2). This antiviral response was impaired under the experimental conditions in lung tissue of HDM-sensitized mice. Reduced levels of IFN-β (267 vs. 500 pg/mg), MCP-1 (28 vs. 87 ng/mg), TNF-α (1.2 vs. 2.1 ng/mg), and IP-10 (18 vs. 41 ng/mg) were observed. RV-induced release of IFN-α was comparable between PCLS of non-sensitized with 112 pg/mg and HDM-sensitized mice with 125 pg/mg.

RV-infected PCLS were also treated with rupintrivir. The amount of virus measured by TCID_50_ or PCR was not reduced by treatment with rupintrivir (data not shown). Rupintrivir showed no reduction of RV-induced cytokine release in PCLS of non-sensitized mice. Rupintrivir reduced IP-10, IL-4, IL-6 and IL-10 close to baseline levels in lung tissue of HDM-sensitized mice ex vivo, but had no effect on the interferon response (IFN-α, IFN-β, and IFN-γ). UV-inactivated RV did not trigger antiviral cytokine secretion (IFN-α, IFN-β, and IFN-γ) compared to uninfected medium control (Fig. 2).

**Fig. 3:**
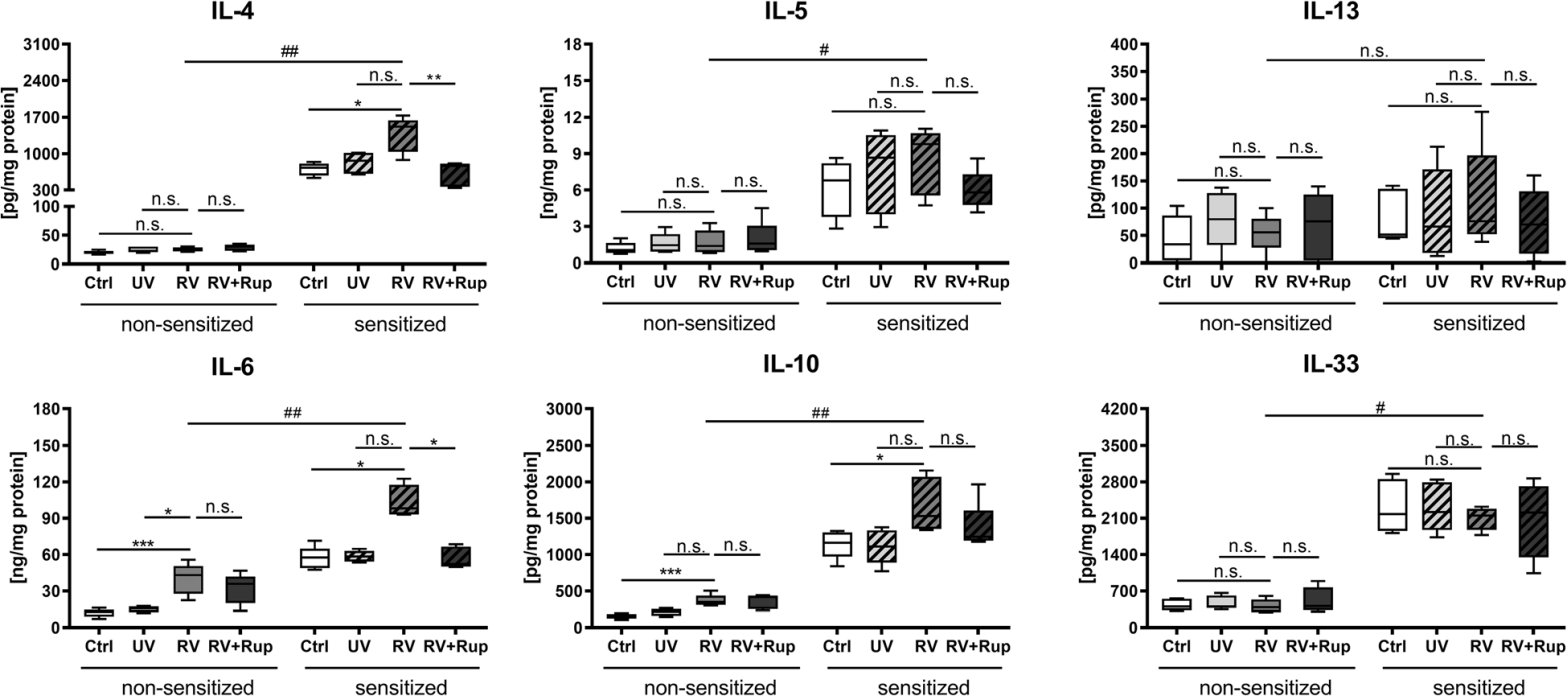
Rupintrivir treatment reduces the exaggerated pro-inflammatory cytokine IL-6 and T_H_-2 cytokine IL-4 induced by RV (rhinovirus) in lung tissues of HDM-sensitized mice. PCLS from non-sensitized and HDM-sensitized mice were infected with 10^5^ TCID_50_/mL of UV-inactivated RV (UV) or RV, or RV in combination with 100 nM rupintrivir (Rup) for 48 h. Uninfected slices were used as a control (Ctrl). All data are shown as box whisker plots (mean to max), *n* = 5 mice with two technical replicates (duplicate wells with 2 PCLS each), statistical analysis performed for non-sensitized or HDM-sensitized group with ** *p* ≤ 0.01; *** *p* ≤ 0.001; **** *p* ≤ 0.0001; according to Friedman with Dunn’s multiple comparison post-hoc test. Means of RV infected PCLS from non-sensitized and HDM-sensitized animals were considered statistically significant with # *p* ≤ 0.05 and ## *p* ≤ 0.01 according to the Mann–Whitney U test. n.s. = not significant

Exaggerated secretion of the T_H_-2 cytokine IL-4 was observed ex vivo in tissue from HDM-sensitized mice upon RV infection, whereas RV had no effect on IL-4 secretion in PCLS from non-sensitized mice (Fig. 3). Rupintrivir attenuated the IL-4-induced exaggeration in RV infected PCLS of HDM-sensitized mice by reducing the IL-4 levels from 1520 to 763 pg/mg. The overall levels of IL-5 increased in PCLS of HDM-sensitized mice, but were not exaggerated by the RV infection. IL-13 levels remained unaltered in both groups. RV induced a pro-inflammatory response in both non-sensitized and sensitized lung tissues, resulting in enhanced secretion of IL-6 in non-sensitized tissue (16 vs. 43 ng/ mg), however, it was more pronounced in tissue of HDM-sensitized mice (58 vs. 98 ng/mg). The anti-inflammatory cytokine IL-10 was elevated in PCLS from both groups upon viral infection with 218 vs. 348 pg/mg in the non-sensitized group, and 1112 vs. 1530 pg/mg in the HDM-sensitized group. The overall levels of IL-6 and IL-10 were exaggerated in RV-infected slices from the HDM-sensitized group. Rupintrivir showed no effect on IL-6 or IL-10 in tissue from non-sensitized animals, however, it resulted in reduced levels of IL-6 (98 vs. 52 ng/mg), and IL-10 (1530 vs. 1246 pg/mg) in tissue of HDM-sensitized mice. IL-33 remained unchanged upon viral infection in both groups when compared to the corresponding UV-inactivated control, although the overall level of IL-33 was higher in the HDM-sensitized group (Fig. 3). UV-inactivated RV did not trigger pro-inflammatory or T_H_-2 cytokine secretion compared to uninfected medium control.

**Fig. 4:**
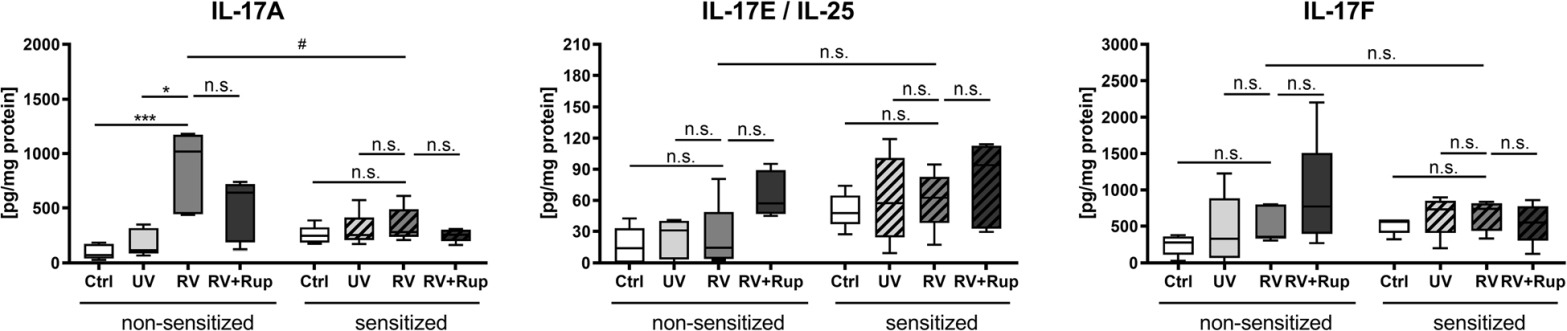
RV-induced secretion of IL-17A is attenuated in PCLS of HDM-sensitized mice. PCLS from non-sensitized and HDM-sensitized mice were infected with 10^5^ TCID_50_/mL of UV-inactivated RV (UV) or RV, or RV in combination with 100 nM rupintrivir (Rup) for 48 h. Uninfected slices were used as a control (Ctrl). All data are shown as box whisker plots (mean to max), *n* = 5 mice with two technical replicates (duplicate wells with 2 PCLS each), statistical analysis performed for non- sensitized or HDM-sensitized group with ** *p* ≤ 0.01 according to Friedman with Dunn’s multiple comparison post-hoc test. Means of RV infected PCLS from non-sensitized and HDM-sensitized animals were considered statistically significant with # *p* ≤ 0.05 according to the Mann–Whitney U test. n.s. = not significant

During viral infection, IL-17A was also enhanced in PCLS isolated from non-sensitized mice, however, it remained at a baseline in lung tissue derived from HDM-sensitized mice. Rupintrivir treatment did not inhibit IL-17A secretion triggered by RV in tissue of non-sensitized animals, although there was a trend. UV-inactivated RV infection did not trigger IL-17 cytokine secretion compared to uninfected medium control (Fig. 4).

## Discussion

Antiviral drugs such as rupintrivir may have an immune-modulatory effect in experimentally induced allergic asthma with subsequent RV infection. We infected lung slices of HDM-sensitized asthmatic mice ex vivo. Our results imply that tissue slices of allergic mice showed a reduced epithelial and antiviral response to RV infection ex vivo. At the same time T_H_-2 cytokines such as IL-4 were increased. Rupintrivir reduced RV-induced cytokines IL-4 and IL-6 in lung slices of asthmatic mice in comparison with slices of untreated asthmatic animals. While the response to RV infection of lung slices of asthmatic mice was expected, the data for the efficacy of rupintrivir in RV-infected lung slices of asthmatic mice are new.

Limitations of our study are mainly due to the ex vivo infection of lung slices. Precision-cut lung slices enable evaluation of the natural tissue response to RV infection. They contain most cell types of lung tissue, including e.g. airway epithelial cells and macrophages [16]. Lung slices of diseased donors show altered histology and often contain inflammatory cells such as eosinophils, neutrophils, macrophages and lymphocytes [17]. However, the connection to blood and lymphoid circulation is missing in PCLS. Cells are unable to migrate from the blood stream into lung tissue and from lung tissue into the lymphoid fluid. As cytokines and chemokines coordinate the migration of cells, these proteins can be used to predict cellular responses in lung slices [18, 19]. A second limitation is that we infected lung slices permanently, without removing the virus inoculum. We were unable to detect any increase of virus based on TCID_50_ or PCR. An explanation could be that the total number of airway epithelial cells is very low in PCLS. The number of virus produced by these few epithelial cells was presumably too low to further increase the initial virus titer. We used interferon-gamma-induced protein 10 (IP-10) as a marker for successful infection [20]. Actively replicating virus induces IP-10 in lung slices whereas UV-inactivated virus is unable to increase IP-10.

Our results show that RV infection ex vivo differs between lung slices obtained from asthmatic and healthy mice. RV induced a normal antiviral response in lung tissue of healthy animals. We observed increased levels of IFN-α, IFN-β, IFN-γ, MCP-1, TNF-α, IP-10, IL-6, IL-10 and IL-17A. In contrast, PCLS of asthmatic mice showed reduced levels of epithelial cytokines type I (IFN-α/β) and NK-cell secreted type II (IFN-γ) IFN response to RV infection. These cytokines act both directly as antivirals as well as indirectly by regulating the activity of natural killer cells, phagocytosis of macrophages, and adaptive immune responses [21]. Infected lung tissue of asthmatic mice also secreted less pro-inflammatory cytokines TNF-α and MCP-1, but produced more T_H_-2 cytokine IL-4. This is in line with results received from peripheral blood mononuclear cells of healthy and asthmatic subjects, where RV infection also increased IL-4 expression in asthmatic subjects [22]. IL-33 and IL-17A levels were elevated in the tissue and BAL of asthmatic mice. Studies have shown elevated levels of epithelial-derived IL-33 in asthmatic patients [23] and mice [24] leading to airway inflammation and hyperresponsiveness [23, 25]. IL-17A is secreted by T_H_-17 cells and is involved in mucosal as well as epithelial host defense [26, 27]. Graser and colleagues showed that IL-17A was responsible for RV clearance in epithelial cells. IL-17A levels were diminished in asthmatic children infected with RV [28]. In accordance with our study, ex vivo infection reduced IL-17A levels in lung slices of asthmatic animals. This indicates that lung tissue of asthmatic mice is capable of reacting to the viral infection but with a reduced capacity of inducing protective immune responses. These results confirm an impaired antiviral response towards RV infection under allergic conditions in vivo [12]. Asthmatic patients experimentally infected with rhinovirus showed more severe disease, increased viral burden and stronger airway inflammation in comparison to infected healthy individuals [29, 30].

Patients with asthma have an unmet need for effective treatment of virus-induced exacerbations. Our research is related to the open question whether antiviral drugs such as rupintrivir exert beneficial effects in asthma patients during exacerbation. Previous studies showed a strong antiviral effect of rupintrivir in vitro against a broad range of clinical RV isolates [8, 31, 32]. Rupintrivir was not toxic in cell culture experiments [8, 10], and did not cause adverse events in healthy volunteers or patients [11, 33]. Nevertheless, it failed to improve symptoms in patients with naturally acquired colds [32]. The efficacy of rupintrivir in asthmatic patients is unknown. In this study, Rupintrivir showed only a minor effect on RV infection in lung slices of healthy animals whereas it inhibited RV-induced exaggerated cytokines IP-10, IL-4, and IL-6 in lung slices of asthmatic animals. IFNs were more or less unchanged.

These data are in line with clinical data showing elevated levels of IL-6 in sputum of asthmatic children during virus-induced exacerbation [34] and IL-4 triggered airway responsiveness in asthmatic patients [35]. Additionally, a study of Massoud and colleagues showed that IL-4 and IL-6 are involved in converting induced regulatory T (T_reg_) cells into T_H_-17 like cells, therefore potentially contributing to asthma severity [36]. Thus, reduced levels of IL-4 and IL-6 by rupintrivir could abolish the ongoing inflammation triggered by T_H_-2 cytokines IL-4 and IL-6 in asthmatic patients.

Our findings raise new questions about different efficacy of antiviral drugs, which may depend on the disease background of the infected individuals. Asthmatic patients may benefit from antiviral interventions whereas healthy individuals do not. Further studies are required in order to understand the biological mode of action of rupintrivir before conducting clinical trials.

## Additional file

Additional file 1. Confocal image analysis and quantification after RV or UV-inactivated RV infection of mouse PCLS. (a)-(d) Tissue slices were stained with 4 μM calcein AM and 4 μM EthD-1 after 48 h of submerged cultivation. Images were examined by confocal laser scanning microscopy and analyzed with IMARIS. Green color shows the cytoplasm of vital cells and red color shows dead cell nuclei (diameter of 4 μm). (e) *n* = 3 independent experiments with three analyzed images per slice (duplicate slices per condition) (One-way ANOVA). Scale bar: 500 μm.

## Data Availability

All data generated or analyzed during this study are included in this published article.

## Abbreviations

DPBS: Dulbecco’s phosphate buffered salt solution
HDM: House-dust mite
IL: Interleukin
IU: Infectious units
PCLS: Precision-cut lung slices
RV: Rhinovirus
TCID_50_: Tissue culture infective dose 50
UV: Ultraviolet
