# Supplementary material for: Rupintrivir reduces RV-induced TH-2 cytokine IL-4 in precision-cut lung slices (PCLS) of HDM-sensitized mice ex vivo
Source: Respir Res. 2019 Oct 22;20:228. doi: 10.1186/s12931-019-1175-y (PMC6805592; doi:10.1186/s12931-019-1175-y)
Supplement: Supplementary file 1 — Confocal image analysis and quantification after RV or UV-inactivated RV infection of mouse PCLS. (a)-(d) Tissue slices were stained with 4 μM calcein AM and 4 μM EthD-1 after 48 h of submerged cultivation. Images were examined by confocal laser scanning microscopy and analyzed with IMARIS. Green color shows the cytoplasm of vital cells and red color shows dead cell nuclei (diameter of 4 μm). (e) n = 3 independent experiments with three analyzed images per slice (duplicate slices per condition) (One-way ANOVA). Scale bar: 500 μm. [file 12931_2019_1175_MOESM1_ESM.docx]

**PCLS remain viable after RV-infection *ex vivo***

Tissue viability maintained 48 h after viral infection with RV. Dead control represents Triton X-100 lysed tissue. Uninfected control, UV-inactivated RV and RV were quantified by Live/Dead staining (Additional file 1).

|   **Control**  **(a)** | 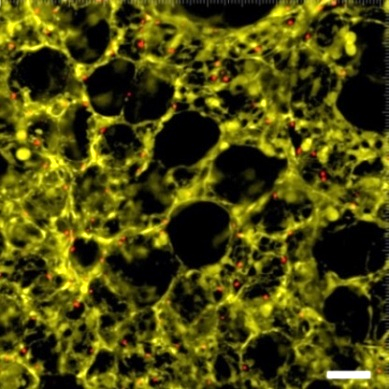  **(b)**  **UV-RV** |   **(e)** |
| --- | --- | --- |
| 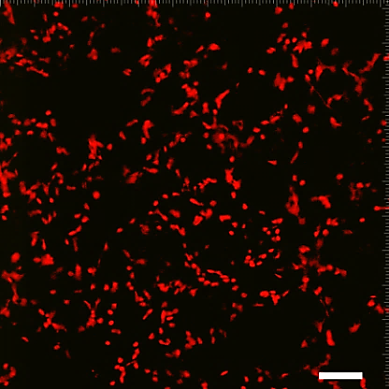  **(c)**  **Dead control** | 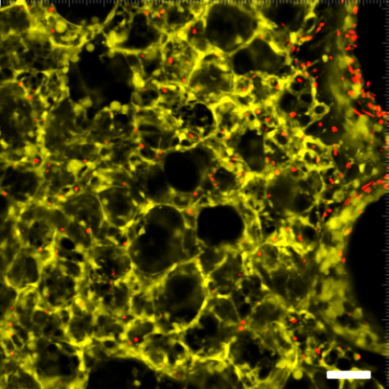  **(d)**  **RV** |  |

**Additional file 1.** Confocal image analysis and quantification after RV or UV-inactivated RV infection of mouse PCLS. (a)-(d) Tissue slices were stained with 4 µM calcein AM and 4 µM EthD-1 after 48 h of submerged cultivation. Images were examined by confocal laser scanning microscopy and analyzed with IMARIS. Green color shows the cytoplasm of vital cells and red color shows dead cell nuclei (diameter of 4 µm). (e) n=3 independent experiments with three analyzed images per slice (duplicate slices per condition) (One-way ANOVA). Scale bar: 500 µm.
